# Prevalence and Risk Factors for Refractive Errors: Korean National Health and Nutrition Examination Survey 2008-2011

**DOI:** 10.1371/journal.pone.0080361

**Published:** 2013-11-05

**Authors:** Eun Chul Kim, Ian G. Morgan, Hirohiko Kakizaki, Seungbum Kang, Donghyun Jee

**Affiliations:** 1 Department of Ophthalmology and Visual Science, Buchon St. Mary’s Hospital, College of Medicine, Catholic University of Korea, Suwon, Korea; 2 Research School of Biology, ARC Centre of Excellence in Vision Science, Australian National University, Canberra, Australia; 3 Department of Ophthalmology, Aichi Medical University, Nagakute, Aichi, Japan; 4 Department of Ophthalmology and Visual Science, Daejon St. Mary’s Hospital, College of Medicine, Catholic University of Korea, Suwon, Korea; 5 Department of Ophthalmology and Visual Science, St. Vincent’s Hospital, College of Medicine, Catholic University of Korea, Suwon, Korea; Zhongshan Ophthalmic Center, China

## Abstract

**Purpose:**

To examine the prevalence and risk factors of refractive errors in a representative Korean population aged 20 years old or older.

**Methods:**

A total of 23,392 people aged 20+ years were selected for the Korean National Health and Nutrition Survey 2008–2011, using stratified, multistage, clustered sampling. Refractive error was measured by autorefraction without cycloplegia, and interviews were performed regarding associated risk factors including gender, age, height, education level, parent's education level, economic status, light exposure time, and current smoking history.

**Results:**

Of 23,392 participants, refractive errors were examined in 22,562 persons, including 21,356 subjects with phakic eyes. The overall prevalences of myopia (< -0.5 D), high myopia (< -6.0 D), and hyperopia (> 0.5 D) were 48.1% (95% confidence interval [CI], 47.4–48.8), 4.0% (CI, 3.7–4.3), and 24.2% (CI, 23.6–24.8), respectively. The prevalence of myopia sharply decreased from 78.9% (CI, 77.4–80.4) in 20–29 year olds to 16.1% (CI, 14.9–17.3) in 60–69 year olds. In multivariable logistic regression analyses restricted to subjects aged 40+ years, myopia was associated with younger age (odds ratio [OR], 0.94; 95% Confidence Interval [CI], 0.93-0.94, *p* < 0.001), education level of university or higher (OR, 2.31; CI, 1.97–2.71, *p* < 0.001), and shorter sunlight exposure time (OR, 0.84; CI, 0.76–0.93, *p* = 0.002).

**Conclusions:**

This study provides the first representative population-based data on refractive error for Korean adults. The prevalence of myopia in Korean adults in 40+ years (34.7%) was comparable to that in other Asian countries. These results show that the younger generations in Korea are much more myopic than previous generations, and that important factors associated with this increase are increased education levels and reduced sunlight exposures.

## Introduction

Refractive errors can lead to visual impairment and ultimately, even blindness [1]. Although they can usually be corrected by wearing glasses or contact lenses, and via surgery, these solutions pose public health challenges and/or economic burdens [2-4]. Uncorrected refractive errors are a major cause of visual impairment [1], and may lead to loss of productivity. In addition, refractive errors are risk factors for various ocular diseases. Myopia, especially high myopia is associated with open-angle glaucoma, rhegmatogenous retinal detachment, cataract, and chorioretinopathy such staphyloma and chorioretinal atrophy [5,6], whereas hyperopia is associated with angle-closure glaucoma [7], and acute ischemic optic neuropathy [8].

Previous epidemiological studies on refractive errors have revealed marked differences between ethnic groups in different parts of the world [9,10]. In particular, the rate of myopia has increased very rapidly in East Asia [1,11-15]. We previously reported an extremely high prevalence of myopia (96.5%) and high myopia (20.6%) in 19-year-old males in urban areas of Korea [12], and a relative high prevalence of myopia (83.3%) and high myopia (6.8%) in a rural area [15]. Because of the limited age range and specific locations of these studies, the results may not be representative of the general Korean population. Comprehensive data on prevalence of refractive errors in Korean adults has not previously been available, and accurate information on the prevalence of refractive errors is important for estimating the need for eye care in this fast-changing health sector in Korea. It may also be useful in elucidating the causes of the current epidemic of myopia, and thus predicting the emergence of problems in less-developed countries as well as potential problems in developed countries that do not have yet an epidemic of myopia.

Although numerous studies have investigated ethnic variations in myopia [13,16-18], most studies have used random sampling of a specific district or city. These studies may therefore not be representative of the population of a given country, and may be limited through the confounding of ethnicity with study site. To our knowledge, only the US National Health and Nutrition Evaluation Study (NHANES) has attempted to use stratified, multistage, clustered sampling methods over a whole nation to describe the prevalence of refractive error, along with many other health parameters [9,10,19]. The Korean National Health and Nutrition Examination Study (KNHANES) uses a similar sampling procedure to give nationally representative data, and, as with the more recent rounds of US NHANES, uses noncycloplegic refraction to measure refractive error.

Risk factors for refractive errors have been extensively investigated in previous population based studies [20-23]. Among these, education is a strong and consistent risk factor for myopia [12,23-25]. Our previous study on Korean young adults also showed that, of several variables, including height, only education was associated with myopia in multivariable analysis [12]. However, the effect of education on myopia in a population-based sample of Korean adults has not yet been evaluated. 

Recently, time spent outdoors has been identified as a potential protective factor against myopia in children [21,26]. Moreover, time spent outdoors, rather than any specific activity performed outside, appears to be the important factor, which suggests that exposure to bright sunlight might play a critical role in the prevention of myopia. Hence, there is a greater need to evaluate outdoor activity associated factors, such as sunlight exposure time. In addition, since most studies evaluating outdoor activity and myopia have been carried out on children, the effect of sunlight exposure time on myopia as adults has only been rarely studied.

In the present study, we examined the prevalence of refractive errors in a representative Korean population aged 20 years old or older, selected using a stratified, multistage, clustered sampling method. We also evaluated associated risk factors, focusing on education and sunlight exposure times.

## Subjects and Methods

### Study Population

This study was based on data acquired in the Korean National Health and Nutrition Examination Survey (KNHANES). The KNHANES is an ongoing population-based cross- sectional, and nationally representative survey conducted by the Division of Chronic Disease Surveillance, Korean Center for Disease Control and Prevention. The survey consists of a health interview, a nutritional survey, and a health examination survey. The survey collected data via household interviews and by direct standardized physical examinations conducted in a specially equipped mobile examination center. In the KNHANES, the sample design and size are designed so that the survey results can be generalized to the whole Korean population.

Each year, 4,000 households in 200 enumeration districts were selected by a panel to represent the civilian, non-institutionalized South Korean population using stratified, multistage clustered sampling based on National Census Data. All members of each selected household were asked to participate in the survey, and the participation rate between 2008 and 2011 ranged from 77.8% to 82.8%.

All participants were given information about the study, and gave written informed consent. The study design followed the tenets of the Declaration of Helsinki for biomedical research and was approved by the Institutional Review Board of the Catholic University of Korea in Seoul, Korea.

### Data collection

Refraction without cycloplegia was measured using an auto-refractor (KR-8800^®^; Topcon, Tokyo, Japan) by ophthalmology residents or ophthalmologists. Refraction measurements were converted into spherical equivalents, calculated as the spherical value plus half of the astigmatic value. Myopia was defined as ≤ -0.50 diopters (D) and as ≤ -1.0 D , high myopia was defined as ≤ -6.0 D, hyperopia was defined as ≥ +0.5 D and as ≥ +2.0 D, and emmetropia was defined as between -0.5 D and +0.5 D. Astigmatism was defined as > +1.0 D. A slit-lamp examination (BM 900; Haag-Streit AG, Koeniz, Switzerland) was performed by ophthalmologists to identify pheudophakic and aphakic patients, who were excluded from the study, and to evaluate cataract status. 

Demographic and socioeconomic information was obtained from a health interview. Height was measured using a wall-mounted measuring scale, and weight was measured with the individual wearing light clothing without shoes in kilograms using calibrated electronic scales. Body mass index (BMI) was calculated using the universally recognized formula: weight (kg) / height (m)^2^.

The educational level of participants and their parents was classified on a scale of 0 to 4, as follows: 0, no education; 1, elementary education; 2, middle school graduate; 3, high school graduate; and 4, university graduate or higher. Economic status was classified into four quartiles, which was based on personal annual income. Data on current sunlight exposure time was obtained by selecting a single question from the two questions: under 5 h, or over 5 h a day. Current smoking history was also examined by questionnaire which consisted of yes or no question.

### Statistical analyses

Age- and gender-specific prevalences of myopia, hyperopia and astigmatism were assessed. The analysis of variance or chi-square test was used to compare the demographic characteristics. Logistic regression models were used to determine the risk factors for myopia, hyperopia and astigmatism in subjects aged 40 years or older. All variables for logistic regression analysis were examined for multicollinearity, and only variables with a variance inflation factor less than 10 were used. Sampling was weighted by statisticians by adjusting the oversampling and non-response rate [27]. Analyses were performed using the Statistical Package for the Social Sciences (SPSS ver. 18.0; SPSS, Inc., Chicago, IL, USA).

## Results

Of 23,392 eligible subjects aged over 20 years, refractive error was measured for 22,562 subjects (96.5%). Of these, subjects with pseudophakic or aphakic eyes (1,206 eyes) were excluded. Thus, 21,356 subjects were included the analysis for prevalence of myopia. Analysis of risk factors for refractive errors was restricted to the subjects aged 40+ years (n = 14,285).

The demographic characteristics of 21,356 subjects enrolled in the study are summarized by refractive status in Table 1. Subjects with myopia were more likely to be younger, taller, female, smokers and have higher education levels, higher parental education levels, higher economic status, and shorter sun exposure times than those without myopia (*P* < .001, respectively). 

**Table 1:** Demographic and clinical characteristics according to myopia (< -0.5 D) and hyperopia (> 0.5 D) in the Korean National Health and Nutrition Examination Survey 2008-2011.

**Characteristics**	**Myopia**	**Emmetropia**	**Hyperopia**	***P***	**Participants**	**Pseudophakic**	***P***
	**(n = 10,262)**	**(n = 5,928)**	**(n = 5,166)**		**(n = 21,356)**	**(n = 1,206)**	
**Age (yrs)**	41.0 ± 13.4	49.2 ± 13.7	63.3 ± 10.9	<.001	48.6 ± 15.7	71.1 ± 10.1	<.001
**Female (%)**	57.3	55.5	57.8	<.001	56.9	63.1	<.001
**Height (cm)**	164.1 ± 9.1	162.3 ± 9.2	158.6 ± 9.3	<.001	162.3 ± 9.4	156.0 ± 9.8	<.001
**Weight (Kg)**	63.4 ± 12.4	62.7 ± 11.2	60.2 ± 11.7	<.001	62.4 ± 11.6	57.7 ± 10.2	<.001
**Body mass index**	23.5 ± 3.5	23.7 ± 3.2	23.8 ± 3.1	<.001	23.6 ± 3.4	23.6 ± 3.2	.941
**Education level (%)**				<.001			<.001
No school	0.9	1.7	5.6		2.3	12.5	
Elementary school	9.2	24.4	47.4		22.3	55.3	
Middle school	7.0	13.9	16.6		11.2	10.1	
High school	40.4	37.0	20.4		34.6	14.7	
University	42.5	24.0	10.0		29.5	7.4	
**Father's education (%)**				<.001			<.001
No school	8.0	15.6	26.5		14.2	32.7	
Elementary school	32.9	44.3	52.0		40.3	50.4	
Middle school	18.8	14.9	8.5		15.4	7.5	
High school	25.7	16.9	8.8		19.5	6.9	
University	14.7	8.3	4.3		10.6	2.5	
**Mother's education (%)**				<.001			<.001
No school	16.1	29.7	50.4		27.7	61.9	
Elementary school	39.8	44.0	39.6		40.9	31.9	
Middle school	18.4	12.6	5.0		13.8	2.9	
High school	20.6	11.3	4.1		14.3	2.6	
University	5.0	2.4	1.0		3.4	0.6	
**Economic status**				.004			0.263
1st quartile (low)	23.9	24.9	24,6		24.3	25.5	
2nd quartile	24.2	26.8	25.7		25.3	25.8	
3rd quartile	26.1	23.4	25.8		25.3	24.6	
4th quartile	25.8	24.9	24.0		25.1	24.2	
**Sun exposure time (> 5 hours) (%)**	15.2	23.7	30.0	<.001	21.1	27.1	<.001
**Current smoker (%)**	20.9	20.6	16.7	<.001	19.4	13.9	<.001

Data are expressed as means ± standard deviation or frequency (%).

The ANOVA was used for continuous variables, and the Chi-square test was used for categorical variables.

**Figure 1:**
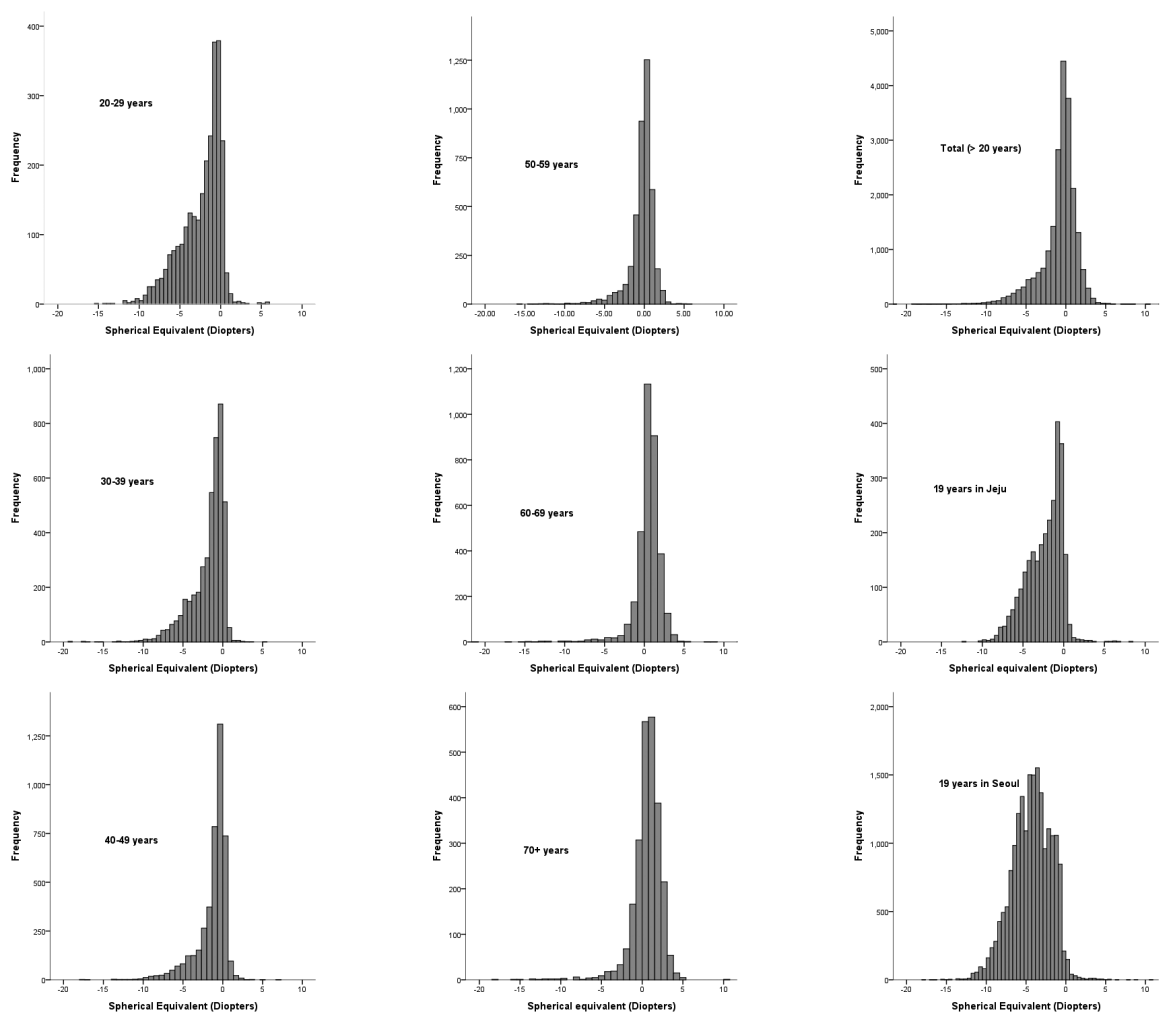
Distribution of refractive error in Korean adults in Korean representative population. Distribution was presented according to the age groups, and compared with those of previous studies for Korean 19 years old population in Seoul and Jeju.

Distribution of refractive error in each age group is shown in Figure 1. Kurtosis and skewness for distribution of refractive error in each age groups was presented in Table 2. For comparative purposes, the distributions of mean SER in 19 year-old males in Seoul and the rural area of Jeju Province are also shown. In the older age groups, kurtosis was high and skewness was low, with a hyperopic peak of refraction, which is the classical picture of the distribution of refractive error [28]. In the younger people, there is a much less kurtosis and much greater spread in the distribution, with a pronounced skew towards myopia. This is seen in an exaggerated form in the data from Seoul. Decreased kurtosis and greater skew progressively appears in the younger age-groups. 

**Table 2:** Kurtosis and skewness for distribution of refractive error in Korean adults in Korean representative population.

	19 yrs (Seou)	19 yrs (Jeju)	20-29 yrs	30-39 yrs	40-49 yrs	50-59 yrs	60-69 yrs	> 70 yrs
Kurtosis	0.43	0.51	1.08	5.87	9.54	19.84	25.68	17.18
Skewness	-0.32	-0.60	-1.04	-1.88	-2.40	-3.10	-3.39	-2.74

Kurtosis and skewness were presented according to the age groups, and compared with those of previous studies for Korean 19 years old population in Seoul and Jeju.

**Figure 2:**
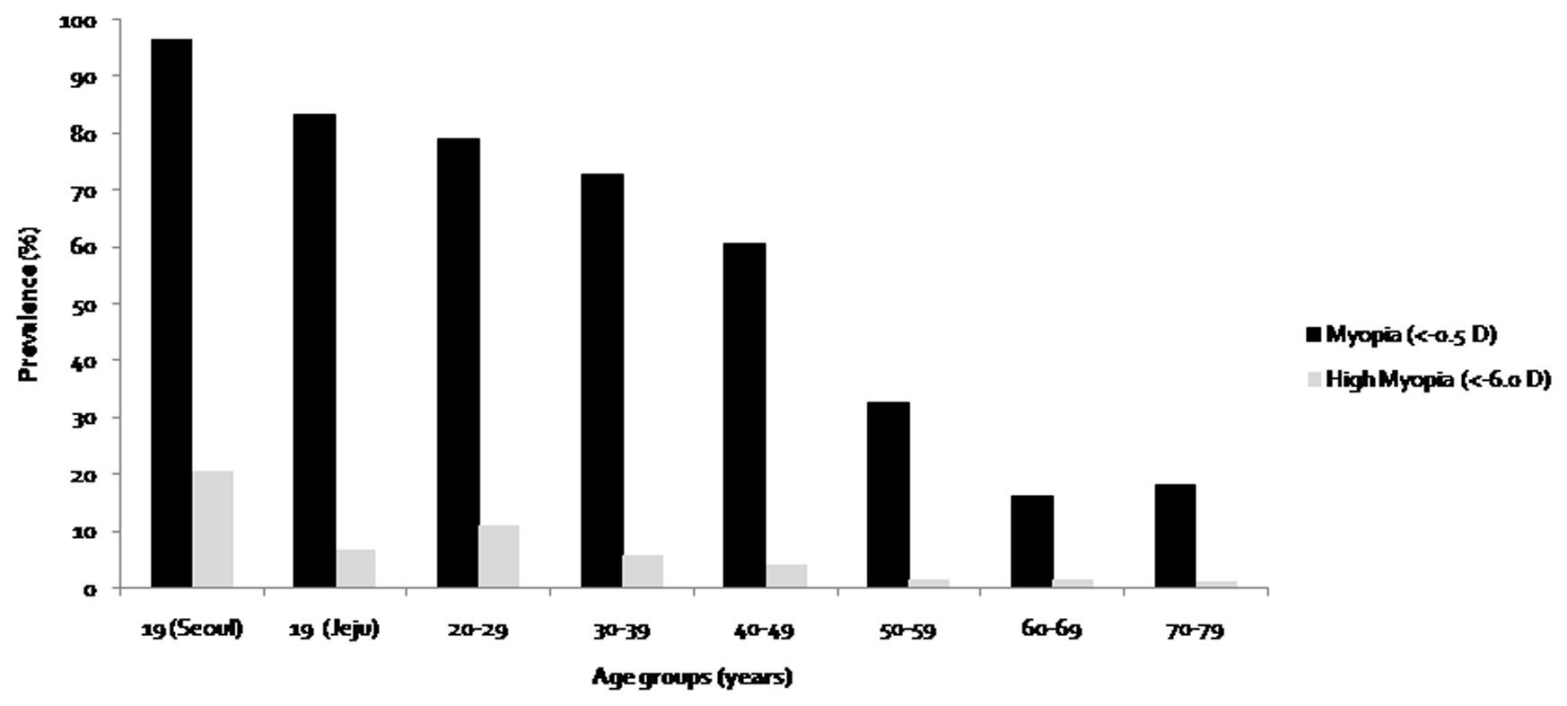
Prevalence of myopia (<-0.5 Diopters [D]) and high myopia (<-6.0 D) in Korean adults in Korean representative population. Prevalence was presented according to the age groups, and compared with those of previous studies for Korean 19 years old population in Seoul and Jeju.

The overall prevalence of myopia and high myopia was 48.1% (CI, 47.4–48.8) and 4.0% (CI, 3.7–4.3), respectively. There is no significant difference in prevalence of myopia between males and females (*p* = 0.142). The prevalence of myopia was markedly higher at 78.9% (CI, 77.4–80.4) in 20–29 year olds compared to 16.1% (CI, 14.9–17.3) in 60–69 year olds (Table 3). Prevalences of myopia and high myopia according to age were shown in Figure 2. Once again, data from our previous studies in Seoul and Jeju Province are included for comparative purposes. The prevalence of myopia in Korean adults showed a biphasic pattern, declining with age and then increasing in the higher aged group after 60-69 years old. However, when analysis was done after excluding subjects with cataract, this biphasic pattern was not shown, and prevalence of myopia declined even in the oldest age group (15.4% in 60-69 years old and 10.5% in 70+ years old).

**Table 3:** Prevalence of myopia, hyperopia, emmetropia, and astigmatism of Korean adults stratified according to age and gender in the Korean National Health and Nutrition Examination Survey 2008-2011.

**Age**	**Number**	**Myopia (<-0.5 D)**	**Myopia (<-1.0 D)**	**High myopia (<-6.0 D)**	**Hyperopia (> 0.5 D)**	**Hyperopia (> 2.0 D)**	**Astigmatism (> 1.0 D)**
**Both genders**							
20-29	2690	2123 (78.9, 77.4-80.4)	1731 (64.4, 62.6-66.2)	294 (10.9, 9.7-12.1)	76 (2.8, 2.2-3.4)	13 (0.5, 0.2-0.8)	762 (28.3, 26.6-30.0)
30-39	4381	3189 (72.8, 71.5-74.1)	2385 (54.3, 52.8-55.8)	251 (5.7, 5.0-6.4)	122 (2.8, 2.3-3.3)	7 (0.2, 0.1-0.3)	972 (22.1, 20.9-23.3)
40-49	4318	2623 (60.7, 59.2-62.2)	1746 (40.4, 38.9-41.9)	177 (4.1, 3.5-4.7)	239 (5.5, 4.8-6.2)	14 (0.3, 0.1-0.5)	1065 (24.6, 23.3-25.9)
50-59	4056	1322 (32.6, 31.2-34.0)	769 (18.9, 17.7-20.1)	63 (1.5, 1.1-1.9)	1184 (29.2, 27.8-30.6)	88 (2.2, 1.7-2.7)	1248 (30.7, 29.3-32.1)
60-69	3438	555 (16.1, 14.9-17.3)	335 (9.7, 8.7-10.7)	46 (1.3, 0.9-1.7)	2018 (58.7, 57.1-60.3)	366 (10.6, 9.6-11.6)	1654 (47.9, 46.2-49.6)
70+	2473	450 (18.2, 16.7-19.7)	311 (12.6, 11.3-13.9)	26 (1.1, 0.7-1.5)	1527 (61.7, 59.8-63.6)	451 (18.2, 16.7-19.7)	1589 (64.3, 62.4-66.2)
> 20	21356	10262 (48.1, 47.4-48.8)	7277 (34.0, 33.4-34.6)	857 (4.0, 3.7-4.3)	5166 (24.2, 23.6-24.8)	939 (4.4, 4.1-4.7)	7290 (34.0, 33.4-34.6))
> 40	14285	4950 (34.7, 33.9-35.5)	3161 (22.1, 21.4-22.8)	312 (2.2, 2.0-2.4)	4968 (34.8, 34.0-35.6)	919 (6.4, 6.0-6.8)	5556 (38.8, 38.0-39.6)
*P* for trend		P < .001	P < .001	P < .001	P < .001	P < .001	P < .001
**Males**							
20-29	1148	894 (77.9, 75.5-80.3)	734 (64.0, 61.2-66.8)	100 (8.7, 7.1-10.3)	30 (2.6, 1.7-3.5)	3 (0.3, 0.0-0.6)	370 (32.2, 29.5-34.9)
30-39	1828	1312 (71.8, 69.7-73.9)	985 (53.7, 51.4-56.0)	94 (5.1, 4.1-6.1)	50 (2.7, 2.0-3.4)	2 (0.1, 0.0-0.2)	444 (24.2, 22.2-26.2)
40-49	1883	1157 (61.4, 59.2-63.6)	778 (41.3, 39.1-43.5)	83 (4.4, 3.5-5.3)	101 (5.4, 4.4-6.4)	6 (0.3, 0.1-0.5)	443 (23.5, 21.6-25.4)
50-59	1713	591 (34.5, 32.2-36.8)	367 (21.4, 19.5-23.3)	30 (1.7, 1.1-2.3)	458 (26.7, 24.6-28.8)	31 (1.8, 1.2-2.4)	494 (28.8, 26.7-30.9)
60-69	1545	248 (16.1, 14.3-17.9)	146 (9.4, 7.9-10.9)	15 (1.0, 0.5-1.5)	876 (56.7, 54.2-59.2)	128 (8.2, 6.8-9.6)	741 (47.7, 45.2-50.2)
70+	1082	179 (16.5, 14.3-18.7)	119 (11.0, 9.1-12.9)	2 (0.2, 0-0.5)	666 (61.6, 58.7-64.5)	159 (14.7, 12.6-16.8)	715 (66.1, 63.0-68.9)
> 20	9199	4381 (47.6,46.6-48.6)	3129 (34.0, 33.0-35.0)	324 (3.5, 3.1-3.9)	2181 (23.7, 22.8-24.6)	329 (3.6, 3.2-4.0)	3207 (34.8, 33.8-35.8)
> 40	6223	2175 (35.0, 33.8-36.2 )	1410 (22.6, 21.6-23.6)	130 (2.1, 1.7-2.5)	2101 (33.8,32.6-35.5)	324 (5.2, 4.6-5.8)	2393 (38.3,37.1-39.5)
*P* for trend		P < .001	P < .001	P < .001	P < .001	P < .001	P < .001
**Females**							
20-29	1542	1229 (79.7, 77.7-81.7)	997 (64.7, 62.3-67.1)	194 (12.6, 10.9-14.3)	46 (3.0, 2.1-3.9)	10 (0.6, 0.2-1.0)	392 (25.4, 23.2-27.6)
30-39	2553	1877 (73.5, 71.8-75.2)	1400 (54.7, 52.8-56.6)	157 (6.1, 5.2-7.0)	72 (2.8, 2.2-3.4)	5 (0.2, 0.0-0.4)	528 (20.6, 19.0-22.2)
40-49	2435	1466 (60.2, 58.3-62.1)	968 (39.7, 37.8-41.6)	94 (3.8, 3.0-4.6)	138 (5.7, 4.8-6.6)	8 (0.3, 0.1-0.5)	622 (25.5, 23.8-27.2)
50-59	2343	731 (31.2, 29.3-33.1)	402 (17.1, 15.6-18.6)	33 (1.4, 0.9-1.9)	726 (31.0, 29.1-32.9)	57 (2.4, 1.8-3.0)	754 (32.0, 30.1-33.9)
60-69	1893	307 (16.2, 14.5-17.9)	189 (10.0, 8.6-11.4)	31 (1.6, 1.0-2.2)	1142 (60.3, 58.1-62.5)	238 (12.6, 11.1-14.1)	913 (48.2, 45.9-50.5)
70+	1391	271 (19.5, 17.4-21.6)	192 (13.9, 12.1-15.7)	24 (1.7, 1.0-2.4)	861 (61.9, 59.3-64.5)	292 (21.0, 18.9-23.1)	874 (62.8, 60.3-65.3)
> 20	12157	5881 (48.4, 47.5-49.3)	4148 (34.0,33.2-34.8)	533 (4.4, 4.0-4.8)	2985 (24.6, 23.8-25.4))	610 (5.0, 4.6-5.4)	4083 (33.5, 32.7-34.3)
> 40	8062	2775 (34.4, 33.4-35.4)	1751 (21.7, 20.8-22.6)	182 (2.3, 2.0-2.6)	2867 (35.6, 34.6-36.6)	595 (7.4, 6.8-8.0)	3163 (39.1, 38.0-40.2)
*P* for trend		P < .001	P < .001	P < .001	P < .001	P < .001	P < .001

Prevalence is expressed as percentage and 95% confidence interval.

Although the overall prevalence of hyperopia (>0.5D) was 24.2% (CI, 23.6–24.8), the age-specific prevalence was lowest at 2.8% (CI, 2.2–3.4) in the 20–29 year olds and much higher at 58.7% (CI, 57.1–60.3) in the 60–69 year olds (Table 3). The overall prevalence of astigmatism was 34.0% (CI, 33.4–34.6), increasing from 22.1% (CI, 20.9–23.3) in the 30–39 year olds to 47.9% (CI, 46.2–49.6) in 60–69 year olds (Table 3).

Education levels by age and gender in the representative Korean population are shown in Table 4. The proportion of those with high education levels (≥ high school graduate) was very much higher in 30-39 year olds (96.9%) than in 70+ year olds (16.2%). Younger age groups were not considered, since many were still completing their education. In addition, the proportion of those with low education levels (≤ elementary school graduate) was very much lower in 30-39 year olds (0.8%) than in 70+ year olds (74.7%). Height was taller in 20-29 years olds than in 70+ year olds (P for trend <0.001; Table 5), whereas the proportion of those with current longer sunlight exposure times (> 5 h) was lower in 20-29 year olds than in 70+ year olds (P for trend <0.001; Table 5).

**Table 4:** Educational levels according to age and gender in a representative Korean population.

	**20-29 years**	**30-39 years**	**40-49 years**	**50-59 years**	**60-69 years**	**70+ years**
**Both gender**	(n = 2690)	(n = 4457)	(n = 4318)	(n = 4056)	(n = 3438)	(n = 2473)
No Education (%)	0.0	0.0	0.1 (0.0-0.2)	0.5 (0.3-0.7)	4.7 (4.0-5.4)	12.7 (11.4-14.0)
Elementary School (%)	0.5 (0.2-0.8)	0.8 (0.5-1.1)	6.0 (5.3-6.7)	29.6 (28.2-31.0)	51.6 (49.9-53.3)	62.0 (60.1-63.9)
Middle School (%)	1.7 (1.2-2.2)	2.2 (1.8-2.6)	11.6 (10.6-12.6)	23.1 (21.8-24.4)	17.3 (16.0-18.6)	9.1 (8.0-10.2)
High School (%)	52.1 (50.2-54.0)	41.4 (40.0-42.8)	46.4 (44.9-47.9)	30.6 (29.2-32.0)	18.6 (17.3-19.9)	10.2 (9.0-11.4)
University (%)	45.7 (43.8-47.6)	55.5 (54.0-57.0)	35.9 (34.5-37.3)	16.2 (15.1-17.3)	7.9 (7.0-8.8)	6.0 (5.1-6.9)
**Male**	(n = 1166)	(n = 1845)	(n = 1883)	(n = 1713)	(n = 1545)	(n = 1082)
No Education (%)	0.0	0.0	0.0	0.4 (0.1-0.7)	1.6 (1.0-2.2)	4.7 (3.4-6.0)
Elementary School (%)	0.3 (0.0-0.6)	0.8 (0.4-1.2)	4.3 (3.4-5.2)	19.2 (17.4-21.0)	34.9 (32.5-37.3)	49.9 (46.9-52.9)
Middle School (%)	1.6 (0.9-2.3)	2.4 (1.7-3.1)	8.7 (7.4-10.0)	21.8 (19.9-23.7)	23.0 (20.9-25.1)	14.9 (12.8-17.0)
High School (%)	62.8 (60.0-65.6)	37.0 (34.8-39.2)	41.0 (38.8-43.2)	32.9 (30.8-35.0)	26.9 (24.7-29.1)	17.8 (15.5-20.1)
University (%)	35.3 (32.6-38.0)	59.9 (57.7-62.1)	46.0 (43.7-48.3)	25.7 (23.7-27.7)	13.6 (11.9-15.3)	12.7 (10.7-14.7)
**Female**	(n = 1560)	(n = 2612)	(n = 2435)	(n = 2343)	(n = 1893)	(n = 1391)
No Education (%)	0.0	0.0	0.2 (0.0-0.4)	0.6 (0.3-0.9)	7.3 (6.1-8.5)	18.9 (16.8-21.0)
Elementary School (%)	0.7 (0.3-1.1)	0.9 (0.5-1.3)	7.1 (6.1-8.1)	36.8 (34.8-38.8)	64.2 (62.0-66.4)	70.5 (68.5-72.9)
Middle School (%)	1.7 (1.1-2.3)	2.1 (1.6-2.6)	13.5 (12.1-14.9)	24.1 (22.4-25.8)	13.0 (11.5-14.5)	4.7 (3.6-5.8)
High School (%)	44.2 (41.7-46.7)	44.8 (42.9-46.7)	50.7 (48.7-52.7)	28.9 (27.1-30.7)	12.2 (10.7-13.7)	4.8 (3.7-5.9)
University (%)	53.5 (51.0-56.0)	52.2 (50.3-54.1)	28.5 (26.7-30.3)	9.6 (8.4-10.8)	3.3 (2.5-4.1)	1.2 (0.6-1.8)

Prevalence is expressed as percentage and 95% confidence interval.

**Table 5:** Height and sunlight exposure time according to age and gender in a representative Korean population.

	**20-29 years**	**30-39 years**	**40-49 years**	**50-59 years**	**60-69 years**	**70+ years**
**Height (cm)**						
Both gender	(n = 2690)	(n = 4457)	(n = 4318)	(n = 4056)	(n = 3438)	(n = 2473)
	166.9 ± 9.1	165.3 ± 8.6	163.5 ± 8.3	161.0 ± 8.7	159.2 ± 8.8	155.7 ± 10.0
Male	(n = 1166)	(n = 1845)	(n = 1883)	(n = 1713)	(n = 1545)	(n = 1082)
	174.3 ± 7.0	172.9 ± 6.1	170.6 ± 5.7	168.3 ± 6.1	166.3 ± 5.5	164.0 ± 6.7
Female	(n = 1560)	(n = 2612)	(n = 2435)	(n = 2343)	(n = 1893)	(n = 1391)
	161.4 ± 5.9	159.8 ± 5.5	157.9 ± 5.3	155.7 ± 5.9	153.4 ± 6.4	149.2 ± 6.9
**Sunlight exposure time (> 5h, %)**						
Both gender	(n = 2690)	(n = 4457)	(n = 4318)	(n = 4056)	(n = 3438)	(n = 2473)
	13.6 (12.3-14.9)	11.4 (10.5-12.3)	16.3 (15.2-17.4)	24.0 (22.7-25.3)	33.7 (32.1-35.3)	34.5 (32.6-36.4)
Male	(n = 1166)	(n = 1845)	(n = 1883)	(n = 1713)	(n = 1545)	(n = 1082)
	21.3 (19.8-22.8)	19.0 (17.2-20.8)	24.5 (22.6-26.4)	32.2 (30.0-34.4)	40.4 (38.0-42.8)	40.4 (37.5-43.3)
Female	(n = 1560)	(n = 2612)	(n = 2435)	(n = 2343)	(n = 1893)	(n = 1391)
	7.9 (6.9-8.9)	5.8 (4.9-6.7)	9.9 (8.7-11.1)	17.9 (16.3-19.5)	28.2 (26.2-30.2)	29.9 (27.5-32.3)

Table 6 shows the risk factors for myopia, hyperopia and astigmatism. In a multivariable regression analysis, the prevalence of myopia was inversely associated with the age (OR, 0.93; CI, 0.93-0.94), and longer sun exposure time > 5 hours a day (OR, 0.84; CI, 0.76-0.93) in Korean adults. Education levels of university or higher (OR, 2.31; CI, 1.97–2.71) and high school (OR, 1.36; CI, 1.19–1.56) were significantly associated with myopia compared to elementary school education or less. Education levels of parents or height were not significantly associated with myopia.

**Table 6:** Associations with myopia, hyperopia and astigmatism in a representative Korean population aged 40+ years.

**Characteristics**	**Myopia (<-0.5 D)**				**Hyperopia (<0.5 D)**				**Astigmatism (>1.0 D)**			
	**Age and Gender Adjusted OR**	***P***	**Multivariable** **analysis**	***P***	**Age and Gender Adjusted OR**	***P***	**Multivariable analysis**	***P***	**Age and Gender Adjusted OR**	***P***	**Multivariable analysis**	***P***
**Age (yrs)**	0.93 (0.92-0.93)	<.001	0.94 (0.93-0.94)	<.001	1.11 (1.10-1.11)	<.001	1.10 (1.10-1.10)	<.001	1.06 (1.06-1.06)	<.001	1.06 (1.05-1.06)	<.001
**Gender (female)**	0.96 (0.89-1.04)	.337	1.10 (0.95-1.26)	.195	1.13 (1.04-1.22)	.003	1.01 (0.87-1.17)	.926	1.05 (0.97-1.12)	.220	0.96 (0.84-1.10)	.568
**Height (cm)**	1.01 (1.00-1.02)	.001	1.00 (1.00-1.01)	.280	0.99 (0.99-1.00)	.034	0.99 (0.99-1.00)	.327	0.99 (0.98-1.00)	.001	0.99 (0.99-1.00)	.043
**Education level (%)**		<.001		<.001		<.001		<.001		.489		.717
< Elementary school	reference		reference		reference		reference		reference		reference	
Middle school	1.00 (0.88-1.14)	<.001	1.01 (0.87-1.16)	.947	1.11 (0.99-1.24)	.085	1.13 (0.99-1.29)	.081	0.93 (0.84-1.04)	.206	0.96 (0.84-1.09)	0.48
High school	1.48 (1.32-1.65)	<.001	1.36 (1.19-1.56)	<.001	0.79 (0.71-0.88)	<.001	0.79 (0.69-0.91)	0.01	0.94 (0.85-1.04)	.224	0.99 (0.87-1.12)	.862
University	2.68 (2.37-3.04)	<.001	2.31 (1.97-2.71)	<.001	0.53 (0.46-0.62)	<.001	0.55 (0.46-0.66)	<.001	0.93 (0.82-1.05)	.220	1.04 (0.89-1.21)	.644
**Father's education (%)**		<.001		.015		.001		.079		.050		.203
No school	reference		reference		reference		reference		reference		reference	
Elementary school	0.98 (0.88-1.10)	.768	0.89 (0.78-1.02)	.104	1.22 (1.02-1.25)	.038	1.16 (1.02-1.32)	.026	1.04 (0.86-1.24)	.705	0.87 (0.77-0.98)	.024
Middle school	1.25 (1.08-1.45)	.003	1.04 (0.87-1.25)	.645	0.96 (0.81-1.13)	.609	1.06 (0.87-1.30)	.539	0.90 (0.76-1.07)	.217	0.89 (0.75-1.06)	.206
High school	1.48 (1.27-1.73)	<.001	1.11 (0.92-1.35)	.271	0.92 (0.77-1.10)	.366	1.08 (0.87-1.34)	.479	0.90 (0.74-1.10)	.304	0.95 (0.79-1.14)	.574
University	1.78 (1.48-2.14)	<.001	1.11 (0.87-1.41)	.399	0.79 (0.63-0.99)	.037	0.89 (0.67-1.19)	.463	0.98 (0.81-1.20)	.869	0.93 (0.73-1.17)	.519
**Mother's education (%)**		<.001		.631		.168		.219		.142		.414
No school	reference		reference		reference		reference		reference		reference	
Elementary school	1.07 (0.98-1.17)	.159	0.95 (0.85-1.07)	.387	1.05 (0.96-1.16)	.280	1.05 (0.93-1.18)	.403	0.94 (0.86-1.02)	.158	1.01 (0.91-1.13)	.831
Middle school	1.30 (1.12-1.52)	.001	0.94 (0.78-1.13)	.498	0.89 (0.74-1.08)	.242	0.99 (0.78-1.25)	.955	0.96 (0.82-1.12)	.611	1.01 (0.83-1.22)	.956
High school	1.66 (1.38-1.99)	<.001	1.01 (0.80-1.89)	.938	0.83 (0.66-1.06)	.139	1.17 (0.88-1.57)	.272	1.17 (0.97-1.42)	.095	1.25 (0.99-1.58)	.067
University	2.24 (1.54-3.25)	<.001	1.24 (0.81-1.89)	.323	1.04 (0.66-1.64)	.872	1.76 (1.06-2.94)	.030	1.05 (0.71-1.54)	.813	1.11 (0.72-1.71)	0.644
**Economic status**		<.001		.128		.045		.154		<.001		.001
1st quartile (low)	reference		reference		reference		reference		reference		reference	
2nd quartile	0.92 (0.82-1.02)	.104	0.88 (0.78-0.99)	.038	1.06 (0.95-1.19)	.277	1.02 (0.89-1.16)	.754	0.91 (0.82-1.00)	.060	0.94 (0.84-1.06)	.308
3rd quartile	1.07 (0.96-1.18)	.246	0.94 (0.84-1.07)	.346	1.12 (1.00-1.25)	.050	1.14 (1.01-1.30)	.039	0.93(0.84-1.03)	.138	0.95 (0.84-1.06)	.358
4th quartile	1.14 (1.03-1.27)	.014	0.88 (0.78-1.00)	.051	0.96 (0.86-1.08)	.528	1.08 (0.95-1.24)	.233	0.79 (0.71-0.87)	<.001	0.80 (0.71-0.90)	<.001
**Sun exposure time (> 5 h,)**	0.72 (0.66-0.79)	<.001	0.84 (0.76-0.93)	.002	1.08 (0.99-1.18)	.079	0.94 (0.84-1.05)	.290	1.07 (0.99-1.16)	.103	1.02 (0.93-1.13)	.646
Current smoker (%)*	0.90 (0.81-1.00)	.060	0.92 (0.82-1.04)	.175	1.08 (0.96-1.21)	.191	0.03 (0.90-1.17)	.667	1.02 (0.92-1.13)	.659	0.98 (0.87-1.10)	.684

* present over absent

Hyperopia was associated with the age (OR, 1.10; CI, 1.10-1.10), and inversely associated with high education level of high school (OR, 0.79; CI, 0.69-0.91), and university or higher (OR, 0.55 CI, 0.46-0.66). Astigmatism was associated with age (OR, 1.06; CI, 1.05-1. 06), and inversely associated with height (OR, 0.99; CI, 0.98-1.00) and higher levels of economic status (OR, 0.80; CI, 0.71-0.90) compared to lower levels.

## Discussion

The present study showed that the prevalence of myopia was high, particularly in the younger age groups, in Korea, in adults aged 20 years or older. In a comparison with other Asian studies with similar ages, the prevalence of myopia in 40 years or older was 34.7%, which is comparable to that in China (26.7 - 32.3%) [16,17], Singapore (Chinese (38.7%) [29], Malays (30.7%) [30] and Indians (28.0%)[31]), and in India (34.6%) [32], but somewhat higher than in Mongolia (17.2%) [33]. When compared with that of Western countries, the prevalence of myopia is higher than in Latinos (16.8%)[34] and those of European origin in Australia (15.0 - 16.9%) [35,36], comparable to that for African-Americans in the United States (36.5%) [9], but lower than that for whites in the United States (42.6%) [9]. Prevalences of myopia in major population based studies are summarized in Table 7.

**Table 7:** Prevalence of myopia in major population based studies.

**Study**	**Year**	**Country**	**Ethnic**	**Number**	**Age**	**Cycloplegia**	**Definition**	**Myopia**				
								**Overall**	**40-49**	**50-59**	**60-69**	**70+**
**KNHNES^a^**	2008-2011	Korea	Korean	14,285	40+^c^	No	< -0.5	34.7	60.7	32.6	16.1	18.2
**Handan eye study**	2006-2007	China	Chinese	6491	30+	NA	< -0.5	26.7	22.0	13.8	14.4	35.1
**Liwan eye study**	2006	China	Chinese	1405	50+	No	< -0.5	32.3	NA	31.7	31.1	40.0
**Tajimi study**	2008	Japan	Japan	3021	40+	No	< -0.5	41.8	67.8-70.3	42.4-49.6	20.8-22.1	13.5-24.6
**The Tanjong Pagar Survey**	1996	Singapore	Chinese	2000	40+	No	< -0.5	38.7	48.9	26.4	28.5	36.8
**Singapore Malay Eye Study**	2004-2006	Singapore	Malay	2974	40-80	No	< -0.5	30.7	31.0	19.6	16.4	33.6
**Singapore Indian Eye Study**	2007-2009	Singapore	Indian	3400	40+	No	< -0.5	28.0	33.3	23.8	20.3	26.9
**Andhra Pradesh Eye Disease Study**	1996-2000	India	Indian	3642	40+	No	< -0.5	34.6	19.2	38.3	56.0	54.1
**Mongol Eye Study**	1995	Mongol	Mongolian	1800	40+	No	< -0.5	17.2	15.6	12.5	21.4	26.5
**NHNES^b^**	1999-2004	U.S.	Whites	5841	40+^c^	No	< -0.5	42.6	52.6		26.4	
**NHNES^b^**	1999-2004	U.S.	Blacks	2343	40+^c^	No	< -0.5	36.5	44.3		23.9	
**Los Angeles Latino Eye Study**	2000-2003	U.S.	Latinos	5927	40+	No	< -1.0	16.8	14.1-17.9	10.5-13.8	8.7-11.6	13.9-34.2
**Beaver Dam Eye Study**	1988-1990	U.S.	Caucasian	4533	43-84	No	< -0.5	26.2	42.9	25.1	14.8	14.4
**Blue Mountain Eye Study**	1992-1994	Australia	Caucasian	3654	40+	No	< -0.5	15.0	25.4	14.4	11.0	11.5
**Visual Impairment Project**	1986	Australia	Caucasian	4744	40+	No	< -0.5	16.9	23.6	16.3	12.4	12.8

^a^ Korean National Health and Nutritional Examination Survey, ^b^ National Health and Nutritional Examination Survey, ^c^ 40+ was chosen for comparison, although total examination age range was 20+ in KNHNES and U.S. NHNES.

**Figure 3:**
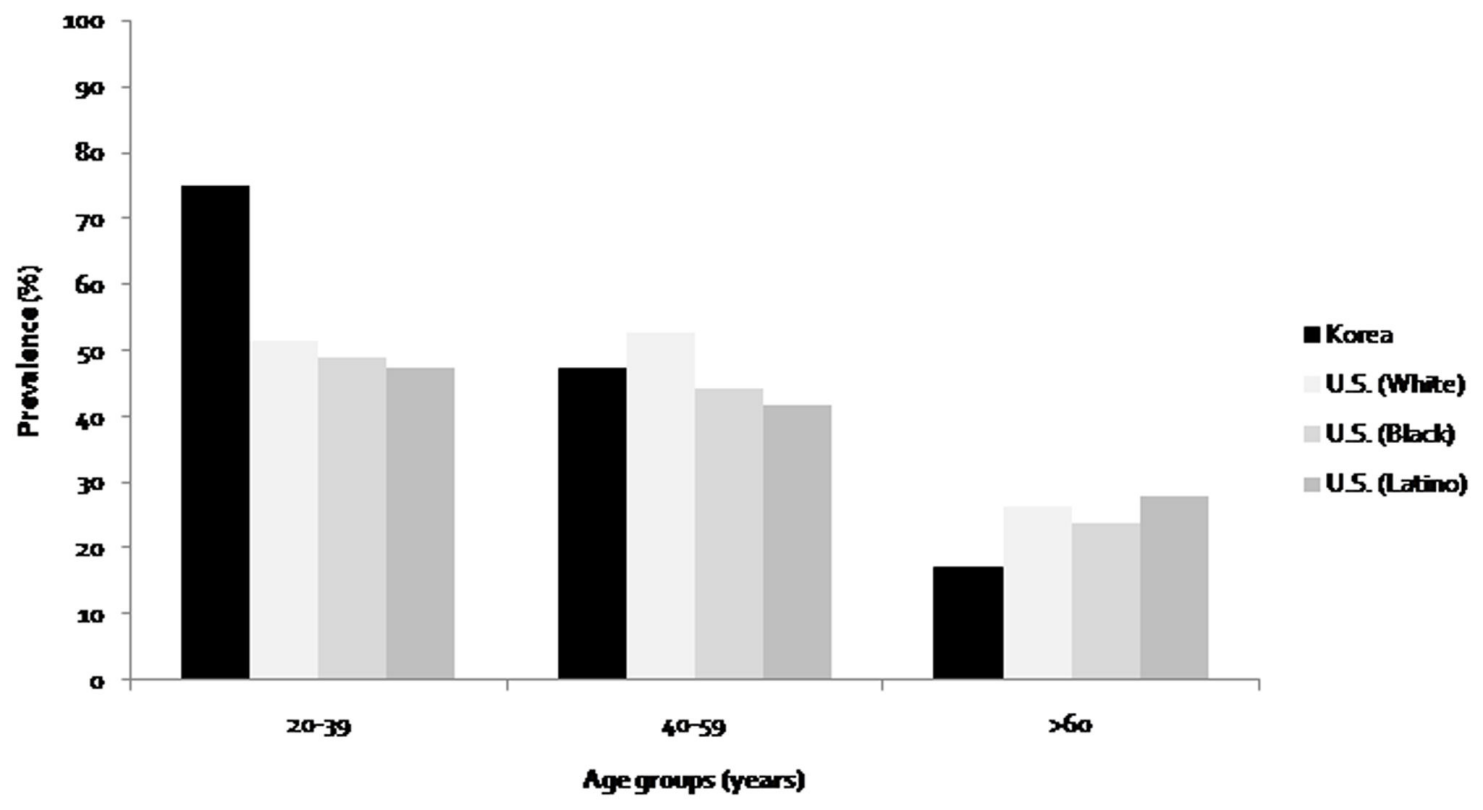
Comparison of prevalence of myopia in Korean National Health and Nutritional Examination Survey (NHANES) with U.S NHANES (Vitale et. al., Prevalence of refractive errors in United States, Archives Ophthalmology 2008).

Age-specific prevalence data are in many ways more meaningful. The prevalence of myopia in 20-39 year-old Korean (75.1%) is higher than in age-matched black (49.0%) and white people (51.3%) in the U.S. NHANES, which used same examination methods, and a similar sampling frame (Figure 3). The prevalence of myopia in 40-49 year-old Koreans (60.7%) is much higher than in similar age groups in other Asian countries. Our data is much higher than Chinese in rural areas (22.0%) [16], Malays in Singapore (31.0) [30], Indians in Singapore (33.3%) [31], Chinese in Singapore (48.9%)[29] and in Indians in India (19.2%) [32]. However, that of Koreans 60 years or older (16.1-18.2%) is comparable or lower than that in other Asian countries (14.4 - 40.0% in Chinese [16], 16.4 - 36.8% in Singapore[29-31], and 54.1 - 56.0% in India [32]). After compared with the other Asian studies without cataract, the prevalence of myopia in those without cataract in 60-69 years (15.4%) and 70+ years (10.7%) is similar to that of Indians and Malays in Singapore [30,31]. Cataract has been found to cause myopic shifts in older adults, which reflects an increased power of the lens rather than increased axial length [30-32]. The difference in the prevalence of myopia between 40-49 and 60-69 years aged groups in Korea (44.0%) was much higher than in Chinese (7.6%) [16], in Singapore (13.0 - 20.4%) [29-31], and Western countries (11.2 - 28.1%)[18,35,36]. This finding suggests that the prevalence of myopia in Korean adults increased more sharply than in other countries.

The reason for rapid increase of myopia in Korean adults is not known. But the history of Korea provides a plausible explanation. After a period of Japanese colonization and the Second World War, Korea was impoverished, but, despite the Korean War, the Republic of Korea has undergone rapid economic development for the last 60 years, documented in a rapid increase in per capita GDP from US$  67 in 1953 to US$  20,050 in 2007 [37]. The increased height seen over this period is further evidence of this economic development. Part of this process of development has been the development of a mass education system. As the questionnaire results show, the proportion of high levels of education (high school or university graduate) in Korean adults increased sharply from 16.2% to 96.9% over 40 years, while those with low education levels (no education or elementary school graduate) decreased from 74.7% to 0.8% over the same period (Table 4). 

The hypothesis that education has played a crucial role is further supported by an Organization for Economic Cooperation and Development (OECD) report concerning change in education over fifty years (accessible at http://www.oecd.org/edu/skills-beyond-school/48642586.pdf). This report shows that rates of educational expansion have varied greatly among countries over recent decades. Korea has made higher education dramatically more accessible, and Korea has been transformed from a country where only a minority of students graduated from secondary school to one in which virtually all students graduate and a high proportion go on to higher education. Thus, Korea has moved from the 21st to the first rank among 25 OECD countries. 

Current sun exposure time in Korean adults was inversely associated with the myopia in our study. Although higher light intensity outdoors reduces the prevalence of myopia in children through the inhibition of eye growth which is caused by the stimulated release of dopamine from the retina or increased focal depth of field by reducing image blur through pupil constriction [21,26], this effect is unlikely to applicable to adults as well, because eyeball elongation tends to decrease after the cessation of body growth. The most likely explanation of this result is that this reflects some continuity in behavior or occupation. The adults who spent more time outside during their childhood, and hence would have been protected from myopia [21], are possibly more likely to spend more time outside as adults, because of their lesser education and the likelihood that they followed occupational paths characteristic for those with less education. A similar inverse relationship between current light exposure and the prevalence of myopia has been reported in Norfolk Island, where those who reported less current time outdoors were less myopic and had higher levels of conjunctival UV autofluorescence, which is a possible semi-cumulative measure of life-time UV exposure [38]. 

The major strength of the present study is the large number of participants (21,356) and the study design using systemic stratified, multistage, clustered random sampling methods. Our study has several limitations. First, the refractive error was measured without cycloplegia, which may lead to the overestimation of the prevalence of myopia, but this is a limitation that it shares with all but one of the major studies on refractive errors in adults, including the US NHANES. Because of this concern, we did not use the data to provide prevalence estimates of refractive error for those aged 5 to 19 years (6,206 subjects). However, as shown by the comparisons to the prevalence of myopia and distribution of refractive error in 19 year-old males in Seoul and Jeju Province measured by cycloplegic autorefraction (Figure 2), there is clear continuity between the results obtained with cycloplegia and those without, suggesting that the patterns are close to those that would be obtained if cycloplegia has been used, although some over-estimation without cycloplegia is likely. This level of agreement is not surprising because, while lack of cycloplegia leads to an over-estimation of myopia, the biggest problems with noncycloplegia refractions concern the under-estimation of hyperopia and the resulting errors in estimation of mean SER [39]. Figure 3 directly compares the results from this study with those from the US NHANES, which has used similar noncycloplegic measurements of refraction. The transition from a lower prevalence of myopia in the older age group in Korea to a much higher prevalence in the youngest age group compared is very clear. This differential pattern of change is unlikely to be due to the lack of cyclopegia. Second limitation is the cross-sectional design, which makes inferring causality difficult. However, in this case we have documented secular changes in education and sunlight exposures which, on the basis of existing evidence on their effects on the development of myopia, could explain the marked differences between age groups. Third, sunlight exposure time was obtained as categorical variable of under 5 hour or over 5 hours a day, not as continuous variables of hours per day. This may limit the evaluation of association of sunlight exposure time with myopia. Finally, smoking variable is a complex variable with many different aspects and just stating it by one yes or no question may not evaluate the enough aspect of this variable such as level of exposure, type of smoking, qualification of smoking, and beginning period of smoking. Unfortunately, these aspects were not fully included in the analysis due to insufficient information about the smoking.

In conclusion, the present study provides the first population-based representative data on refractive errors in the Korean adult population. The prevalence of myopia in 40-49 year old Koreans (60.7%) was very much higher than in other countries, whereas that in 60-69 year olds (16.1%) was comparable to those in many others. This major change may be associated with the rapid economic development of Korea, and its transformation from a poorly educated country to one with amongst the highest educational standards in the world (OECD/PISA). Parallel social changes appear to have reduced the amount of time people are exposed to sunlight outdoors, and myopia in Korea is associated with higher educational level and a lower proportion of those with longer sunlight exposure times (over 5 h per day). In parallel with demonstrated secular changes in these parameters, the prevalence of myopia has increased dramatically in Korea.
